# The importance of aeroelasticity in estimating multiaxial fatigue behaviour of large floating offshore wind turbine blades

**DOI:** 10.1016/j.heliyon.2024.e26017

**Published:** 2024-02-09

**Authors:** Massimo Sirigu, Sara Gigliotti, Davide Issoglio, Giuseppe Giorgi, Giovanni Bracco

**Affiliations:** Marine Offshore Renewable Energy Lab, Politecnico di Torino, Corso Duca degli Abruzzi 24, Torino, 10129, Italy

**Keywords:** Wind energy, Floating offshore wind turbines, Fatigue, Aeroelasticity

## Abstract

The trend toward longer blades in offshore wind turbines poses a significant structural design challenge, given their flexibility and larger load variations. While the study of aeroelastic models of very long blades has gained attention in recent discussions, there is a gap in comprehensive studies examining the impact of different aeroelastic models on fatigue analysis. This study focuses on a comparative evaluation of different aeroelastic models under identical conditions, with a specific focus on multiaxial fatigue. The primary objective is to compare and assess the discrepancies in predicting the lifetime, spatial damage distribution, and critical wind speed conditions. The findings of this study highlight a substantial impact of the aeroelastic model selection on the expected lifetime, revealing durations of 23.2, 3.7, and 1.2 years for the geometrically exact beam theory, Euler-Bernoulli beam, and rigid body assumption. In contrast, the spatial distribution of fatigue damage and critical wind speed conditions remain relatively stable across the models.

## Highlights

• The article provides a comparative analysis of various aeroelastic models for a large wind turbine blade.
• The models include rigid body behaviour, Euler-Bernoulli beam theory, and geometrically exact beam theory.
• A multiaxial fatigue analysis of the composites is conducted for each aeroelastic model.
• The prediction of damage spatial distribution and critical wind speed remains consistent across models.

## Nomenclature

**BECAS**: BEam Cross section Analysis Software
**BEMT**: Blade Element Momentum Theory
**CLD**: Constant Life cycle Diagram
**DEL**: Damage Equivalent Load
**DLC**: Design Load Case
**DNV**: Det Norske Veritas
**ELT**: Expected Life Time
**FEM**: Finite Element Model
**FFT**: Fast Fourier Transform
**GEBT**: Geometrically Exact Beam Theory
**IEA**: International Energy Agency
**NREL**: National Renewable Energy Laboratory
**PI**: Proportional Integral
**SWL**: Sea Water Level
**TSR**: Tip Speed Ratio
**WISPER**: Standardized Fatigue Loading Sequence For Wind Turbine Blades

## 1 Introduction

Offshore wind energy is one of the most promising technologies for renewable energy. The main advantages over onshore wind energy are the access to more consistent winds in open sea, less visual and noise impact, and fewer space constraints to the overall size of the wind farm. The recent growth of offshore wind energy is surprising: in 2020, the global offshore wind power capacity reached 35.3 GW among which 25 GW only in Europe, and it is expected to grow up to 382 GW by the end of 2030 [1].

Thanks to higher and smoother wind power availability, as well as lower environmental and visual constraints, offshore wind turbines are typically larger than onshore [2]: in Europe, the average rated power of installed turbines in 2020 was 7.5 MW. The trend is to manufacture even larger wind turbines, in order to reduce the impact of installation and maintenance costs [1]. Siemens Gamesa recently announced its turbine SG 14-222 DD with a rated power of 14 MW [3] and Vestas published its V236-15.0 MW wind turbine [4]. Large wind turbines have access to higher wind speeds as the hub height is increased, but, at the same time, they experience larger variations in the amplitude of the mechanical stress. In fact, larger diameters cause larger variations in the velocity of the wind due to the wind shear and wind spatial turbulence. Furthermore, the scaling laws, based on the best power fit on reference wind turbines, show that the blade mass increases more rapidly with the diameter compared to the nominal power [5].

In this scenario, accurate evaluation of the aeroelastic dynamics and structural behaviour of large-size wind turbine blades is crucial to avoid unexpected behaviour and premature failures. While the effect of aeroelasticity can normally be neglected for small blades without major errors or risks, it becomes relevant for longer blades: therefore, scientific research is paying more attention to the study of aeroelasticity, since long blades are more compliant and have a lower natural frequency. Consequently, the impact of flutter instability, bend-twist coupling, and buckling phenomena are commonly taken into consideration in the structural design of novel very long blades [6].

Wind turbine blades are typically made of composite materials such as fibreglass and carbon fibre. These materials are prized for their lightweight nature, ease of manufacturing, corrosion resistance, and durability against fatigue over extended periods, surpassing the performance of metal alloys. A relevant topic, connected to the aeroelasticity of the blade, is the fatigue damage of composite materials for offshore wind turbine blades. Historically, the fatigue life of composites is presented as difficult to predict: many factors affect the reliability of the predictions, like the variability of the environmental conditions (wind, waves, temperature, humidity), scarcity of complete fatigue behaviour database of the composites (generally accompanied by a large scatter in the data), non-linear stiffness degradation and damage cumulation, uncertainties in the manufacturing process [6]. However, although an accurate fatigue life prediction in absolute terms is very challenging, fatigue analysis is still useful for design purposes. Both industry and research are incorporating fatigue-driven models into the design of new blades, improving the end-of-life performance of the turbine blades [7].

Methodologies for investigating fatigue in wind turbine blade composites can differ in several aspects, including load assessment, evaluation of internal stress distribution, and the underlying fatigue theory. These methodologies are employed according to the required accuracy, model complexity, and available fatigue data. This paper presents a brief review of fatigue analysis methodologies for offshore wind turbine blades, which are also compared with the methodology herein presented.

Three-dimensional (3D) finite element models (FEM) are commonly used for fatigue analysis [8], [9], [10]. The external loads can be estimated using time domain models, and then properly distributed to the nodes of the FEM. In Hu et al. [11] the distribution of the aerodynamic pressure on the nodes is evaluated using generated by a potential flow analysis software. From the time series of loads, The load cycles for the fatigue analysis are evaluated with the Rainflow algorithm [12], [13]. In Kulkarni et al. [9], deep neural networks are employed to produce the time-variant loads on blade sections. An alternative way to time-variant loads is to use load spectra in the frequency domain. Frequency domain approaches generally use a standardized set of load cycles, called WHISPER, to account for the variation of the mechanical stress over time, which is properly scaled with the actual mechanical stress computed with the static model [8], [14], [15].

Although FEM results are typically very accurate, the required computation time for the evaluation of a complete time history makes this approach inconvenient [16]. For this reason, the use of the quasi-static approach in steady-state conditions is often used. The mechanical stress is evaluated for constant wind speed and the load cycles are the result of the interpolation of time-variant wind with previous quasi-static results. Examples of this kind of approach can be found in Kulkarni et al. [9] and Zhang et al. [17].

As already mentioned, the computation time is the main limit to the use of FEM, and it is difficult to obtain a complete set of load cycles that capture the realistic behaviour of the blade under floating conditions, elastic behaviour, and control system operation. For these reasons, 1D beam theory with 2D FEM section analysis is preferred when the objective is the evaluation of detailed time histories with lower computation time [16]. Interesting results can be achieved using this method: Meng et al. [18] examine the bend-twist coupling effect on blades with varying fibre orientations and its impact on fatigue damage, while Gao et al. [19] investigate how the hydrostatic properties of the floating platform affect the blade fatigue damage. In this paper, the geometrically exact beam theory is applied to compute the internal mechanical stress, demonstrating that also the shear stress is important and multiaxial fatigue analysis must be carried out for large wind turbine blades.

In order to analyze the mechanical stress, the blade is divided into different sections and internal forces and moments are applied according to the position along the blade span. The distribution of the strains and mechanical stress on each section is evaluated in open-source software like BECAS [20], SONATA [21], or commercial software like VABS [22].

Predicting the lifespan of materials requires consideration of the fatigue theory. The selection of the fatigue criterion plays a crucial role in this regard. The linear Goodman method with logarithmic interpolation is commonly used and recommended by DNV standards when detailed material fatigue properties are unavailable [23]. However, DNV advises the utilization of more comprehensive lifecycle diagrams, including a piecewise Goodman diagram, for R values (ratio of the maximum to minimum mechanical stress, see Subsect. 2.4) equal to at least 10, -1, and 0.1.

From the discussion above, it looks clear that there is a variety of alternatives that can be made to model aeroelasticity. Quantitative comparisons among these alternatives are often made to compare power production, thrust, and deformations of the blade. In Sayed et al. [24] a comparative analysis of a CFD (computational fluid dynamics) model and traditional BEMT (blade element momentum theory), both coupled with a Timoshenko flexible beam model for the structure of the blade was conducted, demonstrating a 3.34% difference in the prediction of power production. Hach et al. [25] compare five different tools to aeroelastic blades for simulating the dynamic instability of the blade, finding some discrepancies among the models attributed to unsteady aerodynamics and different results for the torsional deflection of the blade.

In literature, less emphasis is shown on the effect of aeroelasticity on fatigue damage. Qu et al. [26] compares Euler-Bernoulli and geometrically exact beam theory for the DTU 10 MW wind turbine blade. The study evaluates the extreme loads, blade tip displacements, and fatigue results, limited to the longitudinal stress at the blade root.

Due to the limited work in the literature, the present paper aims to fill this gap. A quantitative analysis is carried out to discuss the impact of different aeroelastic models on resulting load cycles and fatigue damage of composites, and to assess the differences in terms of quantitative results, the spatial distribution of multiaxial fatigue damage along the blade span and critical wind speed conditions. The time-domain load analysis is conducted in OpenFAST [27], due to its wide distribution and different aeroelastic modelling options. OpenFAST offers three different aeroelastic modelling options, including rigid body, modal analysis with flapwise and edgewise deflection, and geometrical exact beam theory (GEBT) as implemented in the Beamdyn submodule [28]. Alternative software options, such as HAWC2, Orcaflex, or Bladed, can be utilized in place of Openfast [2]. As an example, HAWC2 employs a distinct approach to aeroelastic models compared to Openfast, notably incorporating the Timoshenko beam, which is not available in OpenFAST.

BECAS, an open-source code developed in Matlab environment and distributed by DTU, is used to evaluate the mechanical stress at the sections [20].

The fatigue study is based on maximum principal stress theory and linear damage cumulation is evaluated using the Palmgren-Miner method and piecewise Goodman diagram. We employ these models due to their extensive utilization in related research [29]. The paper provides a detailed investigation of multiaxial fatigue damage for flexible blades tested on the IEA 15 MW wind turbine. The study focuses on the results of the Beamdyn model, which is the most accurate method to model aeroelastic behaviour. Beamdyn is capable of simulating the bend-twist coupling and the deformations of the blade in six degrees of freedom. The paper presents the load history of internal forces and the distribution of mechanical stress at sections of interest. The results of the fatigue analysis of the rigid body and Elastodyn models are presented to assess the differences.

The remainder of the paper is organised as follows: Sect. 2 explains the methodology, the hypotheses and the design load cases used for the simulations in OpenFAST, the 2D finite element modelling and the fatigue theory; Sect. 3 shows the internal forces from OpenFAST, the mechanical stress from BECAS and the results of the fatigue analysis; Sect. 4 analyses and discusses the results of the fatigue results and presents the strengths and limitations of the methodology and the hypotheses; finally, the conclusions gather the key points of the paper and present final considerations and remarks.

## 2 Materials and methods

The multiaxial fatigue analysis on the floating wind turbine blade is the objective of the work. The case study is the IEA 15 MW reference wind turbine [30], mounted on the Volturn US floating platform [31]. The control system is based on the ROSCO controller [32].

The ROSCO controller was specifically designed for floating offshore wind turbines and rapidly became the reference open-source controller for many projects. It uses common control logic employed in the wind industry, such as tip speed ratio (TSR) tracking control for the generator torque and gain-scheduled PI control for the blade pitch, but it also includes more recent advanced features: an additional control loop for the blade pitch based on the platform pitch velocity feedback, a minimum set for the blade pitch at below-rated wind speed to optimise the power production, and the thrust peak shaving, used to limit the maximum thrust force near the rated wind speed, but at the expense of the energy production.

Three aeroelastic models are implemented in OpenFAST. For the rest of the paper, the three aeroelastic models will be called Beamdyn, Elastodyn, and Rigidbody. The assumptions of the models are explained in Subsect. 2.2.

The overview of the work is highlighted below:

• The internal forces and moments are evaluated along the blade length within OpenFAST 3.4.0 (see Subsect. 2.1), using three aeroelastic models (see Subsect. 2.2).
• The internal forces are implemented in BECAS to obtain the three components of the mechanical stress (longitudinal, transverse, shear stress) on the different composite layers for each section (see Subsect. 2.3).
• The criterion of maximum principal stress is employed to obtain the damage equivalent load (DEL) for different wind speeds (see Subsect. 2.4).
• The DEL curves in the function of the wind speed are interpolated to estimate the expected lifetime (ELT) of the blade for the specific site (see Subsect. 2.5).

### 2.1 OpenFAST model

OpenFAST 3.4.0 is employed to conduct the non-linear, time-domain simulation of the floating wind turbine [27]. FAST is an open-source software developed by NREL and represents the state of the art of wind turbine simulation tools.

OpenFAST includes hydrodynamics, aerodynamics, structural deformation, and mooring submodules, coupled together to obtain the multibody dynamics of the floating wind turbine. The hypotheses of the simulation are resumed in Table 1. The environmental conditions *V*0 (mean wind speed), *Hs* (Significant height) and *Tp* (peak period), described in Table 3, are the input for OpenFAST.

**Table 1:** The hypotheses of simulation in OpenFAST.

Aerodynamics	Blade element momentum theory (BEMT), Aerodynamic pitching moment, Dynamic airflow, skewed wake correction, Beddoes-Leishman unsteady airfoil, Tower influence
Hydrodynamics	Cummins equation, first and second order waves, Quadratic viscous drag
Moorings	Dynamic mooring
Blade	Beamdyn with 1% structural damping and 20th-order polynomial interpolation, Elastodyn with 1% structural damping, Rigid body
Tower	Modal analysis (first two modes)
Wind	Spatial grid dimension: 21x21, wind shear power law exponent: 0.14, Turbulence model: class B
Waves	Jonswap spectrum, unidirectional waves, aligned with the wind

### 2.2 Aeroelastic models

OpenFAST calculates internal forces on the sections considering aerodynamic, gravitational, centrifugal, and gyroscopic forces. Three aeroelastic modelling techniques are compared: the first treats the blade as a rigid body, whereas the second uses the modal analysis in Elastodyn. ElastoDyn uses the Euler-Bernoulli beam theory, considering the first two modes for the flapwise direction and one mode for edgewise direction. The blade is modelled as straight (no prebend included), and the torsion deflection and bend-twist coupling are not included. The third and most accurate method uses geometrically exact beam theory (GEBT) as implemented in Beamdyn [28]. The GEBT considers the deflections (rigid body motion of the sections) in six degrees of freedom, including the torsional deflection and bend-twist behaviour, and can model the initial prebend of the blade. Recent studies [33], [34] have compared the accuracy of GEBT to shell finite element models in predicting the deformations and stresses, demonstrating that GEBT is highly accurate in static and dynamic situations. However, GEBT fails to predict stress in case of buckling phenomena or concentration stresses due to rapid changes in the section properties. The different aeroelastic models affect the loads on the internal structure, and consequently the fatigue damage on the composite materials, in different ways: Primarily, blade compliance plays a role in lowering aerodynamic loads by reducing the apparent wind speed on the airfoil during motion. Moreover, in Beamdyn, the airfoil rotation along its pitch axis results in a reduction of the airfoil's angle of attack. The presence of blade resonance can further impact load cycles, occurring when the external forces' frequencies align with the blade's natural frequencies. This alignment can increase vibration amplitudes and cyclic loading on the blade structure [35].

### 2.3 2D finite element modelling

**Figure 1:**
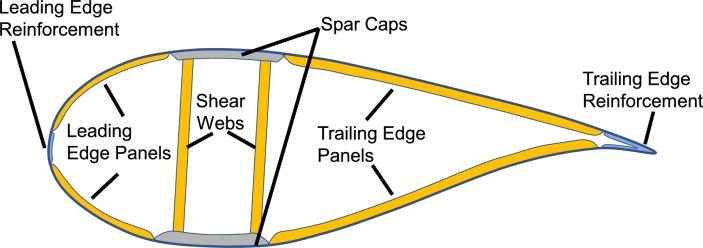
Definition and location of the internal regions of the section of the blade [36].

The section of the blade is defined by several internal regions, represented in Fig. 1 for clarity: the spar caps are the most stressed region because they sustain the flapwise moment; generally the spar caps are made of uniaxial fibreglass or carbon fibre. The trailing edge and the leading edge reinforcements sustain the edgewise moment. The panels connect the spar caps with the leading edge and trailing edge regions; usually the panels have a foam or balsa core to increase the thickness and prevent the local buckling. The shear webs sustain the shear forces and the pitching moment.

Beamdyn requires 6x6 stiffness and mass matrices for each section. The stiffness and mass matrices **K** and **M** are defined as follows:

$$\text{K}=\left[\begin{array}{cccccc} K_{11} & K_{12} & 0 & 0 & 0 & K_{16}\\ K_{21} & K_{22} & 0 & 0 & 0 & K_{26}\\ 0 & 0 & K_{33} & K_{34} & K_{35} & 0\\ 0 & 0 & K_{43} & K_{44} & K_{45} & 0\\ 0 & 0 & K_{53} & K_{54} & K_{55} & 0\\ K_{61} & K_{62} & 0 & 0 & 0 & K_{66} \end{array}\right]$$

$$\text{M}=\left[\begin{array}{cccccc} M & 0 & 0 & 0 & 0 & M_{16}\\ 0 & M & 0 & 0 & 0 & M_{26}\\ 0 & 0 & M & M_{34} & M_{35} & 0\\ 0 & 0 & M_{43} & M_{44} & M_{45} & 0\\ 0 & 0 & M_{53} & M_{54} & M_{55} & 0\\ M_{61} & M_{62} & 0 & 0 & 0 & M_{66} \end{array}\right]$$

The **K** and **M** matrices are symmetric, where $K_{16}$ and $K_{26}$ are the parameters that define the bend-twist coupling behaviour. The linear mass, denoted as *M*, is equal in all three translation directions. BECAS, an open-source code developed by DTU in Matlab environment, serves as both a pre-processor and post-processor in this context: in a pre-process stage, it evaluates the sectional stiffness and mass matrices for Beamdyn; in the post-process, it assesses the strain and stress in the composite materials various layers. BECAS accounts for the composite anisotropic behaviour, as well as the torsional resistance resulting from the airfoil geometry and shear webs [20].

The properties of the sections are derived from the wind turbine ontology file (written in YAML format), published with the FAST model: the ontology file defines the location, width, thickness, and composite layup of the different materials. Linear interpolation of these values based on the ontology file results in 50 defined sections.

**Figure 2:**
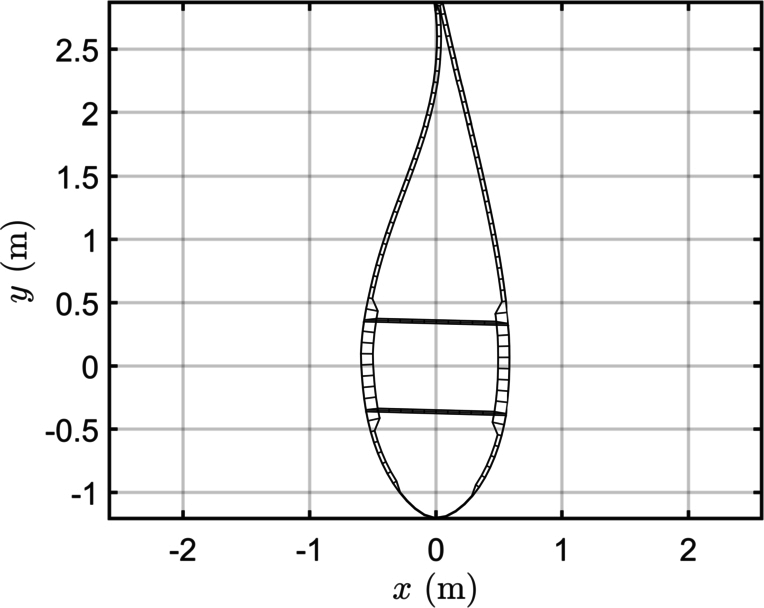
Mesh of the section at 50% of the blade span created in BECAS.

**Figure 3:**
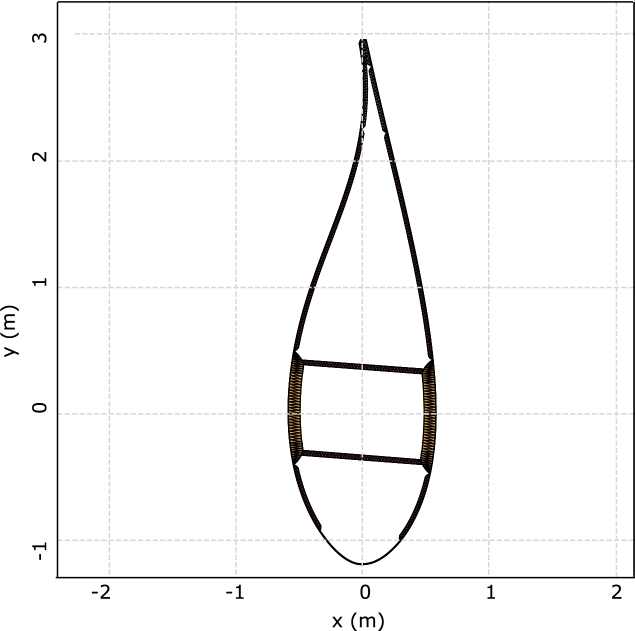
Mesh of the section at 50% of the blade span created in SONATA.

**Figure 4:**
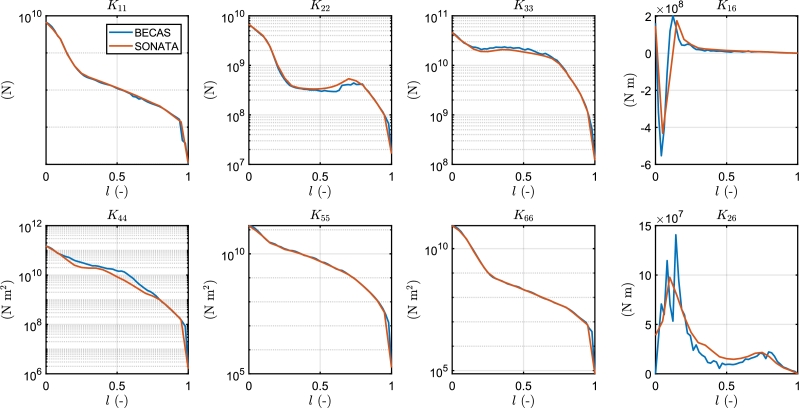
Comparison between the elements of the stiffness matrices obtained with BECAS and SONATA. *l* represents the relative blade span.

Together with the FAST model, the developers published the Beamdyn input parameters, which contain the stiffness and mass matrices of the sections, created with the software SONATA. The match of the stiffness matrices obtained with BECAS and SONATA is the first check to ensure the consistency of the following work, since the stiffness is directly related to the distribution of the mechanical stress on the elements of the sections. Figure 2, Figure 3 display the mesh of the section at 50% of the blade span, as implemented in BECAS, and its comparison with the mesh created in SONATA. The perimeter of the airfoils is discretized with 100 nodes, while each web comprises 12 nodes, and a convergence analysis is performed. Although the mesh in BECAS is similar to the mesh in SONATA, there are some discrepancies. In particular, the thickness of the materials on the trailing edge is different in BECAS, resulting in more separated pieces, whereas SONATA has a more compact trailing edge. This fact explains the differences between the $K_{22}$ and $K_{44}$ components of the two matrices. Furthermore, the connection of the web to the spar cap is modelled differently: in BECAS, the nodes of the webs are directly connected to the spar cap nodes, resulting in a superposition of the areas. This configuration affects the values of $K_{16}$ and $K_{26}$. Fig. 4 illustrates the comparison between the components of the stiffness matrices. BECAS shows more scattering among the different sections of the blade, albeit with a similar pattern. The differences in the mesh can cause variations of up to 10% in the stiffness matrices, especially for the $K_{44}$, $K_{16}$ and $K_{26}$ components. To maintain consistency with other published studies, the original stiffness and mass matrices are employed in the simulations.

### 2.4 Fatigue theory

The conventional fatigue theory assumes that cyclic fluctuations in mechanical stress are responsible for material failure. The material lifespan is expressed in terms of the number of cycles until failure, which depends solely on the amplitude of the stress and not on the cycle frequency. A typical experimental method to determine the material's fatigue properties involves applying a sinusoidal force with a constant amplitude and counting the cycles until failure. To account for the effects of mean stress, several experiments using different specimens are conducted. The ratio of minimum to maximum stress is used to classify the type of test [37], using the parameter *R* defined in Eq. (1).

(1) $$R=\frac{\left(\sigma_{m}-\sigma_{a}\right)}{\left(\sigma_{m}+\sigma_{a}\;\right)}=\frac{\sigma_{min}}{\sigma_{max}}$$

where $\sigma_{m}$ is the mean stress, $\sigma_{a}$ is the amplitude, and consequently $\sigma_{max}$ and $\sigma_{min}$ are the maximum and minimum, respectively. It follows that, for example, R=−1 denotes a sinusoidal function with mean zero, R=0.1 represents pure tensile cycles, and R=10 indicates pure compressive cycles [37]; in general, for alternating stresses, R∈[0,1) represents a pure tensile cycle, R>1 stands for pure compressive cycles, while R<0 indicates alternating tensile-compressive cycles. Wind turbine blades experience various cycles with different mean and amplitude loads. The most commonly used approach for assessing the damage equivalent load (DEL) caused by multiple cycles is the Palmgren-Miner method, which assumes that each load cycle is independent of the others and the cumulative damage is not influenced by the sequence of the load cycles. To assess the number, mean, and amplitude of the load cycles, a Rainflow algorithm according to ASTM E 1049 standard is employed. The maximum value among the three fatigue damage values (longitudinal, transverse, and shear) is used to determine the global damage for each element. To evaluate the expected lifetime (ELT), the DEL is linearly interpolated with the wind speed occurrence [38], as expressed in Eq. (2).

(2) $$ELT=\frac{1}{\sum (3\cdot DEL_{w})\cdot (occ_{w}\cdot 24\cdot 365)}$$

Where $DEL_{w}$ is the *DEL* associated with the wind speed for 20 minutes of simulation and $occ_{w}$ is the occurrence of the wind speed.

The fatigue analysis uses the static material properties from data provided with the openFAST model (shown in Table 2), but detailed fatigue data were not available for the specific materials, such as a definition of the life cycle (S-N) curves for different R values.

**Table 2:** Static strength properties of the materials of the blade.

Material	Longitudi-nal tensile strength (MPa)	Long. compressive strength (MPa)	transverse tensile strength (MPa)	Transv. compressive strength (MPa)	shear strength (MPa)
Carbon fibre	1546	1047	46.6	158	55
Triaxial glass	396	448.9	76.4	174.7	103.4
Biaxial glass	429	70.7	429	70.7	103.4
Uniaxial glass	609.2	474.7	38.1	112.6	18.9

To address this issue, the fatigue behaviour is assumed to be similar to the behaviour of the fibreglass QQ1 and carbon fibre P2B presented in the paper of [39], under the voice “mean fit parameters”. QQ1 is specifically an E-glass/epoxy composite, whereas P2B is a hybrid laminate that integrates carbon fibres with a 0-degree orientation and fibreglass with orientations of ±45 degrees, all within an epoxy matrix. The constant life cycle diagrams (CLD) are adjusted by multiplying the values of the CLD with the ratio of the tensile to compressive strength of QQ1 and P2B to that of the actual material used in the simulation.

**Figure A.17:**
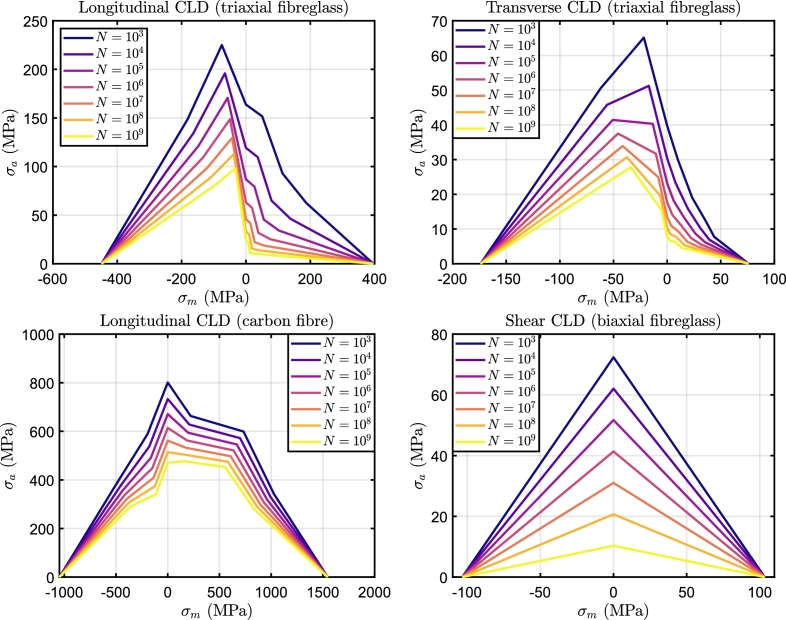
Longitudinal, trasverse and shear stress constant life cycle diagram (CLD) for material of interest. On top, longitudinal and transverse CLD the triaxial fibreglass. On the bottom left, longitudinal stress CLD for the carbon fibre. On the bottom right, shear stress CLD for the biaxial fibreglass.

Shear fatigue data are unavailable in the paper of Samborsky et al. [39]. For the shear CLD of QQ1 and P2B, a linear Goodman diagram is used with an S-N slope of 10, as per the standard [23]. For the biaxial fibreglass, the longitudinal and transverse CLD are obtained by dividing the longitudinal CLD for uniaxial fibreglass by the ratio of the ultimate tensile and the compressive strength of uniaxial fibreglass to the strength of biaxial fibreglass as indicated in Table 2. The relevant resulting CLDs for the composites are shown in Fig. A.17 in the appendix.

### 2.5 Environment conditions

**Figure 5:**
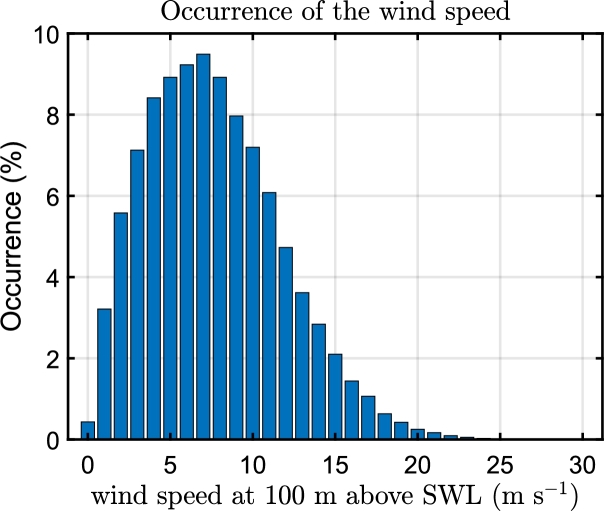
Occurrence of the wind speed in the site of Pantelleria, Italy for ten years of data collection.

To obtain accurate sea state values, a ten-year dataset of wind, wave height, and period scatter data is collected from the shore of Pantelleria, Italy [40]. The scatter data is downloaded from ECMWF ERA5 [41]. The peak period and significant height are then calculated as a weighted average of all sea states within the selected wind speed range. Such an approach eliminates the need for repetitive simulations with the same wind speed, as the effect of the sea state on blade fatigue is negligible. This method is referred to as DLC 1.1 in industry standards [42]. The triplets used in the simulation are summarised in Table 3. In Fig. 5 the occurrence of the wind speed at 100 meters above sea water level (SWL) is illustrated.

**Table 3:** DLC used for the following set of simulation conditions: mean wind speed (*V*), significant wave height (*H*_*s*_), peak period (*T*_*p*_).

*V* (m s^−1^)	*H*_*s*_ (m)	*T*_*p*_ (s)	*V* (m s^−1^)	*H*_*s*_ (m)	*T*_*p*_ (s)
5	1.20	6.24	15	2.64	6.62
7	1.37	6.25	17	3.19	7.06
9	1.59	6.20	19	3.72	7.38
11	1.89	6.24	21	4.31	7.85
13	2.22	6.38	23	4.71	7.95

## 3 Results

**Figure 6:**
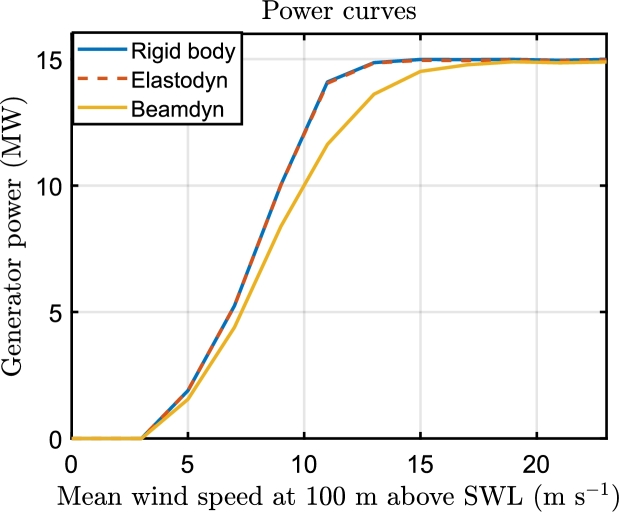
Comparison of the power curve with the aeroelastic models.

This section presents the results of OpenFAST and the fatigue study. The use of GEBT greatly affects the aerodynamic forces of the wind turbine with respect to rigid body assumption or elastic blade using modal analysis. Since the twist deformation decreases the angle of attack of the airfoils, the power curve obtained using Beamdyn is lower than that of the rigid body and Elastodyn assumption, as shown in Fig. 6, and the rated power is reached at higher wind speeds; these results are consistent with prior studies [43]. The prediction of the capacity factor, obtained interpolating the occurrence of the wind speed with the power curves, is 45.74% for rigidbody, 45.67% for Elastodyn and 39.77% for Beamdyn. Since the torsional deformation reduces the loads, the ROSCO controller's inclusion of the thrust peak shaving can be reduced or eliminated to increase energy production. Additionally, as the control system is not designed to consider blade deflections, it necessitates recalibration, which is a potential area of interest for future research.

For each aeroelastic model, a convergence study has been conducted to find the maximum time step that guarantees numerical stability. In Table 4, the time step and the computational time required for each model are reported for 22 minutes of simulation. The computation time of Beamdyn is 9.5 hours for each DLC, which is significantly higher than the Elastodyn and Rigidbody, mainly due to the very small time step required to ensure numerical stability.

**Table 4:** Computational time required for the aeroelastic models for each DLC.

Model	Time step	Computational time	Simulated time
Beamdyn	0.0005 s	9.5 h	22 min
Elastodyn	0.02 s	5.2 min	22 min
Rigidbody	0.02 s	5.1 min	22 min

### 3.1 Internal forces

**Figure 7:**
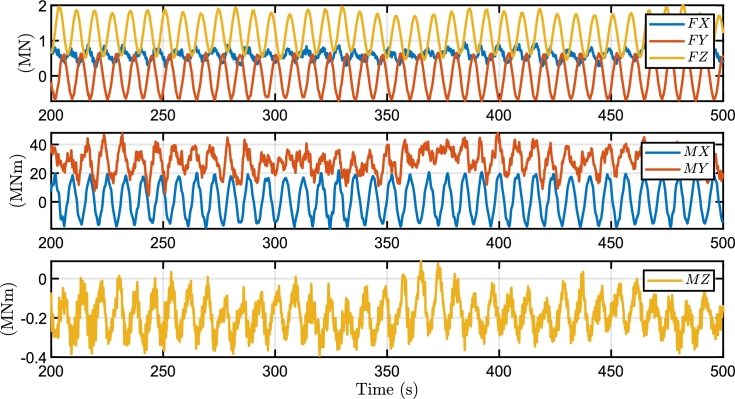
Time history of the load cycles at the blade root in Beamdyn for a wind speed of 15 m s^−1^.

**Figure 8:**
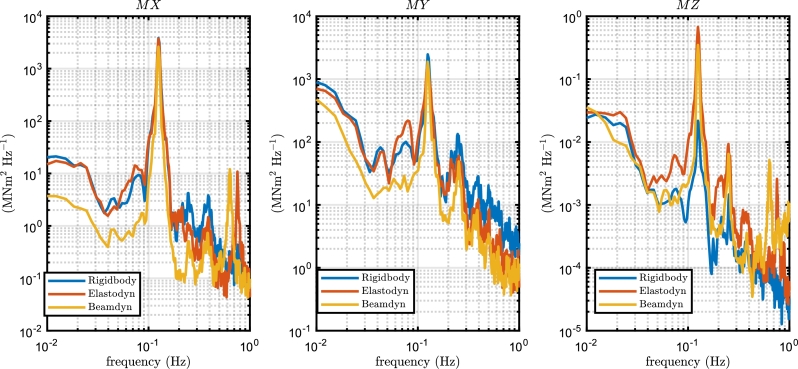
Comparison of the power spectrum density (PSD) of the moments at the blade root associated with the aeroelastic models. The mean wind speed is 15 m s^−1^.

Fig. 7 displays a representative example of time history of loads at the blade root for a mean wind speed of 15 m s^−1^ using Beamdyn assumptions. The first two minutes of simulation are excluded for the fatigue analysis to neglect transient behaviour. The torque moment (*MZ*) is significantly lower than the flapwise and edgewise moments (*MY* and *MX*, respectively). The torque moment includes the aerodynamic pitching moment and the non-diagonal terms of the inertia matrix due to the prebend of the blade and the deformations. Power spectrum density (PSD) curves of the internal moments at the blade root are shown in Fig. 8 for the wind speed of 15 m s^−1^. At above-rated speed, the main peak of *MY* occurs at 0.126 Hz, which corresponds to the nominal rotor speed (7.55 RPM), demonstrating that the primary source of variation of the blade forces is the rotation of the rotor due to wind spatial variability. At lower frequencies, less than 10^−1^ Hz, the wind spectrum is dominant to determine the variation of the internal moments. The effect of the wave forces on the blade, that for a wind speed of 15 m s^−1^ the peak period corresponds to 0.151 Hz, is negligible. The application of Beamdyn results in a decrease in the mean of *MX* and *MY* attributed to its reduced aerodynamic torque caused by the decrease in the airfoil's angle of attack. It is noteworthy that Beamdyn causes a shift in the high-frequency peak from 0.753 Hz in Elastodyn to 0.634 Hz. These high-frequency peaks are attributed to the natural frequency of the blade's structure, coupled to the aerodynamics, as reported also in [31]. These results are consistent with previous works in literature for the floating DTU 10 MW reference wind turbine [26]. Elastodyn and rigid body models show similar behaviour for the internal forces but with higher mean and amplitude values.

### 3.2 Mechanical stress

**Figure 9:**
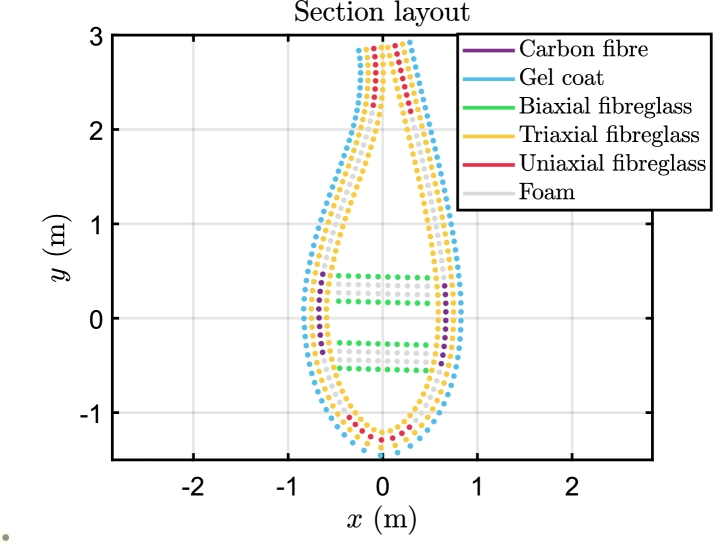
Materials composition of the section at 50% of the blade span.

In BECAS, the mechanical stress is analysed at 50 sections across the blade span. Due to the significant variation in the materials' thicknesses, it may be difficult to distinguish the different layers using the original dimensions; therefore, for clarity, the elements of the nodes are represented as points, regardless of the thickness. The materials that compose the section in the middle are presented in Fig. 9 to show the layers layup.

**Figure A.18:**
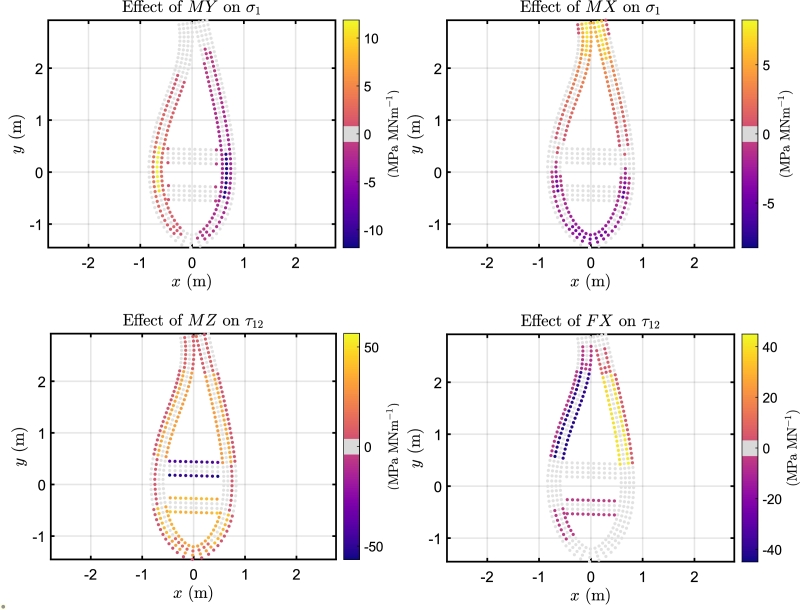
Application of the internal forces and moments on the mechanical stress distribution Each circle represents an element of a mesh. On the top-left, effect of *MY* on the longitudinal stress. On the top-right, effect of *MX* on the longitudinal stress. On the bottom-left, effect of *MZ* on the shear stress. On the bottom-right, effect of *FX* on the shear stress.

The mechanical stress components, namely the longitudinal stress $\sigma_{1}$, transverse stress $\sigma_{2}$, and shear stress $\tau_{12}$, are computed by applying the superposition method to the 6 components of the loads. The computation relies on the assumption of linear elasticity, enabling the differentiation of each load contribution to the stress components. The contribution of the external loads to the stress components is also shown in Fig. A.18 in the appendix for $\sigma_{1}$ and $\tau_{12}$ subjected to *MX* and *MY*.

### 3.3 Fatigue results

The fatigue analysis produces three values of DEL, one for each stress component, for each section of the blade. The ELT is computed considering the maximum value among the DEL of each stress component. The gel coat and foam layers are excluded from the fatigue assessment.

**Figure 10:**
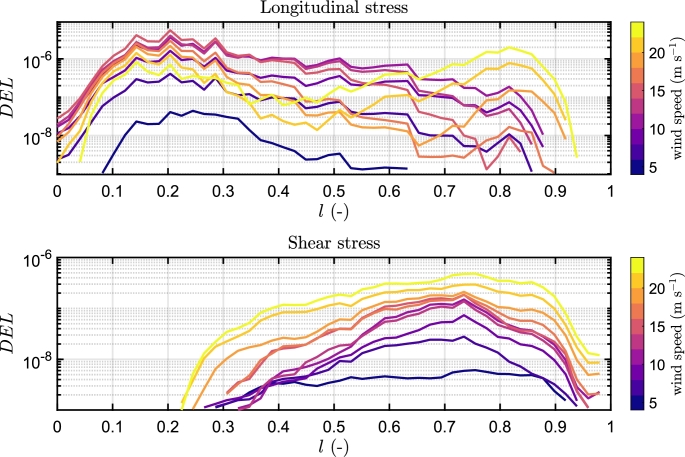
DEL associated with longitudinal and shear stress load cycles along the blade span in Beamdyn. *l* is the relative blade span.

**Figure 11:**
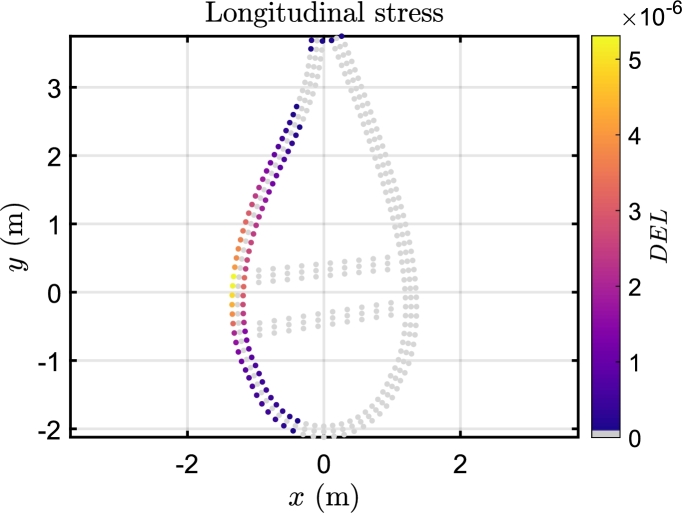
Internal distribution of longitudinal fatigue damage at 20% of the blade span in Beamdyn for the wind speed of 15 m s^−1^. The gel coat layer is not shown.

**Figure 12:**
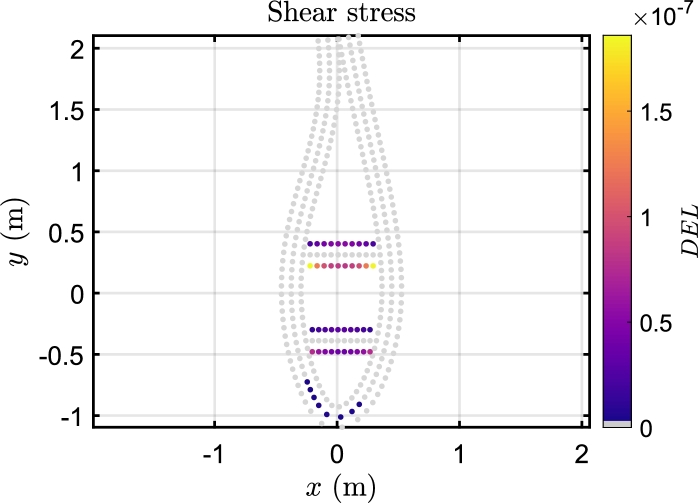
Internal distribution of shear fatigue damage at 73% of the blade span in Beamdyn for the wind speed of 15 m s^−1^.

In Fig. 10, fatigue damage curves depict variations in longitudinal and shear directions across the blade span under different wind speeds under Beamdyn assumptions. The spatial distribution of the DEL is assessed as the maximum damage occurred among the elements of each 2-D mesh of the sections, as can be seen in Fig. 11 for the longitudinal stress and Fig. 12 for the shear stress. The triaxial fibreglass of the outer and inner layers on the pressure side spar cap (tensile stress region) is the critical material for all three aeroelastic models, with maximum DEL due to tensile strength at 20% of the blade span (Fig. 11). The region of the blade at 10-30% of the blade experiences the highest fatigue damage. The critical wind speeds are between 11 and 15 m s^−1^, where the maximum thrust is attained at the rated power, and near the cut-out speed, i.e. between 21 and 23 m s^−1^. The blade root, on the other hand, is less damaged even though the load amplitude is greater, due to the high stiffness necessary for the blade tip clearance, which leads to a higher thickness in this section. However, it should be noted that the blade root in this study is modelled as a solid piece, whereas in real conditions it incorporates the stud bolts.

The fatigue damage on the transverse component is null for every wind speed and section.

In Beamdyn, the critical section for the shear stress is located at 73% of the blade span (Fig. 10). The most stressed regions are the shear webs (Fig. 12).

The ELT, as evaluated in eq. (2), can be used to compare the spatial distribution of the fatigue behaviour of the blade. The ELT is estimated for each element of the mesh by interpolating the DEL curves with the occurrence of the wind speed. The minimum value within the mesh elements is chosen to represent the overall lifespan of the respective section. The ELT value serves as a key indicator for comparison, consolidating an array of load cases into a single, weighted metric. This indicator serves as a weighted representation, factoring in the occurrence of each load case, although this indicator can be affected by different environmental conditions.

**Figure 13:**
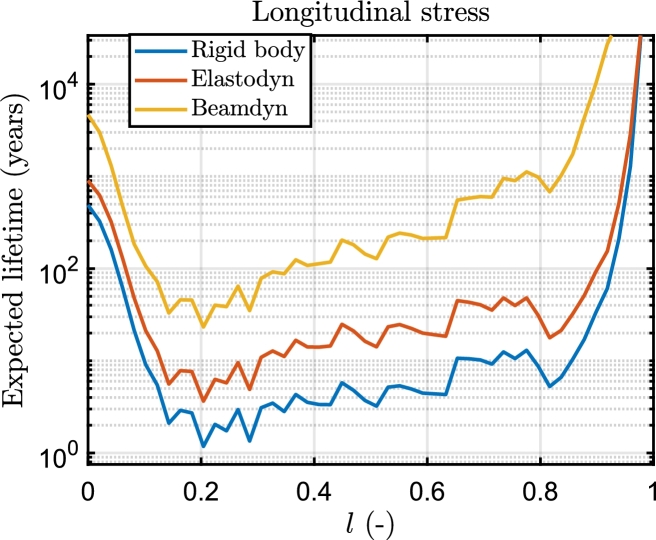
Comparison of the ELT for longitudinal stress of the section at 20% along the blade span for the three aeroelastic models.

**Figure 14:**
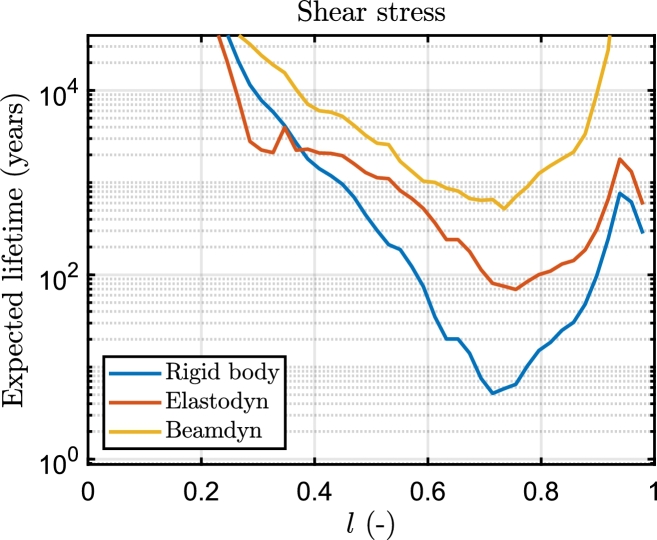
Comparison of the ELT for shear stress of the section at 73% along the blade span for the three aeroelastic models.

**Figure 15:**
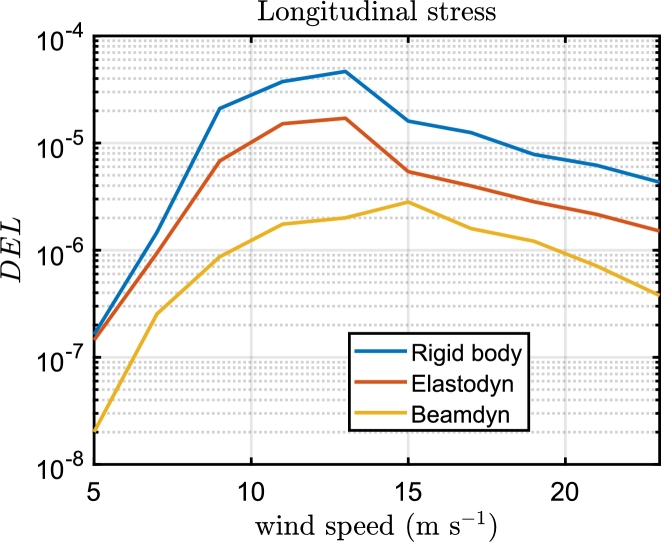
Trends of the DEL for longitudinal stress fatigue damage of the section at 20% of the blade span for the three aeroelastic models.

**Figure 16:**
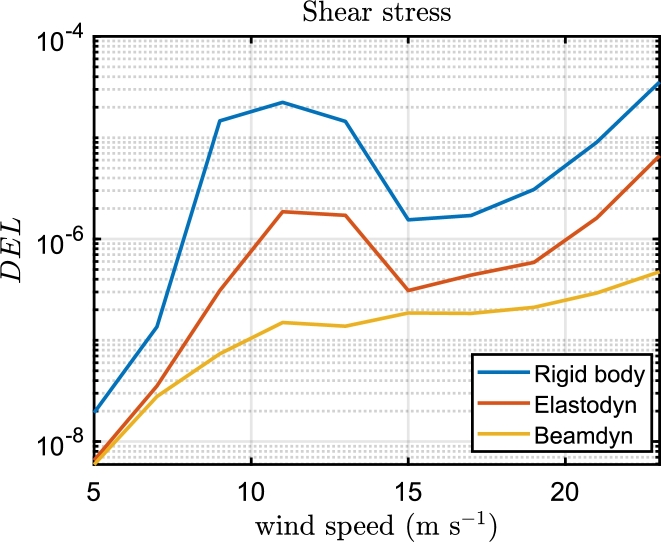
Trends of the DEL for shear stress fatigue damage of the section at 73% of the blade span for the three aeroelastic models.

Figure 13, Figure 14 finally illustrate the comparison among the three models of the ELT across the sections of the blade, for longitudinal and shear stress respectively. As a final point, in Figure 15, Figure 16 the trend of the DEL with the wind speed is shown. The considered sections have the lowest ELT, in particular for the longitudinal stress the section at 20% of the blade span, and for shear stress, the section at 73%.

## 4 Discussion

The ELT of the blade has been estimated using three models, including Beamdyn, Elastodyn, and rigidbody, which yielded life spans of 23.2, 3.7, and 1.2 years for longitudinal stress and 518.9, 69.3 and 5.2 years for the shear stress. The compliance of the blade (flapwise and torsional deflections) helps reduce the peaks of the forces and moments of the aerodynamic loads and significantly impacts the prediction of the ELT. This result is in line with the other works from literature [26]. The low ELT of the blade, which should be higher than 25 years considering different safety factors (see DNVGL-ST-0376 [23]) can be attributed to the poor fatigue resistance of the tensile stress region of the triaxial fibreglass obtained from Samborsky et al. [39], as can be seen in Fig. A.17. Although there are better fibreglass materials available, their complete fatigue behaviour is not adequately documented in the literature. To enhance the accuracy of the ELT prediction, the database for the multiaxial fatigue behaviour of composites needs to be expanded.

Although the prediction of the ELT differs substantially in terms of absolute values, the choice of the three aeroelastic models does not significantly change the spatial distribution of the fatigue damage: in all cases, the analysis reveals that the peak in longitudinal fatigue damage occurs in the triaxial glass at the outer layer of the pressure side spar cap region, located at approximately 20% of the blade. The pattern persists across other sections of the blade, exhibiting a consistent trend. Particularly, in the 20-80% region, the trend approximates a logarithmic shape. Beamdyn registers an even steeper slope on the ELT curve.

On the other hand, the minimum value of ELT for the shear stress is predicted at 73% of the blade span in the biaxial fibreglass of the shear web. The ELT shows a higher damage in the second half of the blade, with negligible damage observed for *l* values below 20%. The trend shows a logarithmic decrement of ELT for *l* within the 20%-80% range. Elastodyn, in contrast to rigidbody and Beamdyn, diverges in its trend, particularly near 30% of *l*. The primary causes of shear fatigue damage in all three models are the shear force *FX* and pitching moment *MZ*.

The trend of the DEL with the wind speed is similar for all three models for longitudinal and shear stress. In particular, the maximum DEL caused by the longitudinal stress is predicted at the rated speed and decreases with higher wind speed, the trend of the DEL caused by the shear stress sharply increases until the rated speed, and then increases more slowly until the cut-out wind speed. In the rigidbody and Elastodyn model, the peak of DEL due to the shear stress shows a reduction of the damage between the nominal wind speed and the cut-out speed, which is not present in Beamdyn.

The use of more complex fatigue failure methods, such as the Tsai-Hill criterion or Puck's criterion, is not expected to significantly change the fatigue damage. The maximum longitudinal stress and shear stress locations do not overlap, and the transverse stress is absent, making it possible to use the maximum stress analysis.

It should be noted that a higher level of detail is required for the evaluation of the connection between the webs and spar caps since the local concentration stresses play an important role. Furthermore, the study does not consider the adhesive layers, which are necessary to connect the webs and spar caps and the two sides of the trailing edge. For these reasons, the authors caution that the fatigue damage study of the shear stress can be considered less reliable compared to the longitudinal stress.

## 5 Conclusions

The study conducted a multiaxial fatigue failure comparison between three aeroelastic models in OpenFAST. The case study is the baseline version of the floating offshore IEA 15 MW wind turbine located in the site of Pantelleria, Italy.

The key findings of the paper can be summarized as follows:

• The platform motion, power curve and load cycles of the wind turbine significantly differ in Beamdyn and the aeroelastic assumptions made by the modal analysis of Elastodyn, which neglects the torsional deformation of the blade, are not adequate for the 15 MW wind turbine.
• It was found that the predicted expected lifetime with the three models is 23.2, 3.7, and 1.2 years for Beamdyn, Elastodyn and Rigidbody respectively.
• The spatial distribution of fatigue damage remains relatively stable across the models, predicting the maximum fatigue damage for longitudinal stress in the triaxial glassfibre at 20% of the blade span.
• The critical wind speed conditions are predicted for all models near the rated wind speed, between 13 and 15 m s^−1^, for longitudinal stress, whereas for shear stress is predicted near the cut-out wind speed (23 m s^−1^).

The methodology presented in this paper can be used to evaluate the performance of the blade under different conditions, such as varying environmental conditions, platform geometries, and control system implementations.

In future studies, it would be valuable to broaden the scope of comparing aeroelastic models in various software tools like HAWC2, Orcaflex, and Bladed, given their inclusion of different aeroelastic models, such as the Timoshenko formulation.

## CRediT authorship contribution statement

**Massimo Sirigu:** Writing – review & editing, Writing – original draft, Formal analysis, Conceptualization. **Sara Gigliotti:** Formal analysis, Data curation. **Davide Issoglio:** Formal analysis, Data curation. **Giuseppe Giorgi:** Writing – review & editing, Supervision, Formal analysis. **Giovanni Bracco:** Supervision, Project administration.

## Declaration of Competing Interest

The authors declare that they have no known competing financial interests or personal relationships that could have appeared to influence the work reported in this paper.
